# Recovering whole-body angular momentum and margin of stability after treadmill-induced perturbations during sloped walking in healthy young adults

**DOI:** 10.1038/s41598-024-54890-4

**Published:** 2024-02-23

**Authors:** Shabnam Shokouhi, Prasanna Sritharan, Peter Vee-Sin Lee

**Affiliations:** Department of Biomedical Engineering, University of Melbourne, Melbourne, VIC 3010 Australia

**Keywords:** Biomedical engineering, Motor control

## Abstract

Although humans are well-adapted to negotiating sloped terrain, balance recovery after a disturbance on slopes is poorly understood. This study investigated how slope affects recovery from unanticipated simulated trips and slips. Eighteen healthy young adults walked on a split-belt treadmill at 1.25 m/s and three slope angles (downhill: − 8°; level: 0°; uphill: + 8°), with slip- and trip-like perturbations applied randomly at heel-strike. We evaluated balance recovery using whole-body angular momentum (WBAM) and perturbation response (PR), for which larger PR values indicate greater deviation of the margin of stability from baseline, therefore, greater destabilisation after perturbation. Overall, trips were more destabilising than slips, producing larger PR and greater range and integral of WBAM across all tested slopes, most significantly in the sagittal plane. Contrary to expectation, sagittal-plane PR post-trip was greatest for level walking and smallest for downhill walking. Heightened vigilance during downhill walking may explain this finding. Recovery strategy in both frontal and sagittal planes was consistent across all slopes and perturbation types, characterized by a wider and shorter first recovery step, with trips requiring the greatest step adjustment. Our findings advance understanding of the robustness of human locomotion and may offer insights into fall prevention interventions.

## Introduction

Human walking exhibits remarkable stability and robustness across diverse and challenging scenarios that are ubiquitous in daily life, including the negotiation of sloped terrain and recovery from disturbances, such as slips and trips, without falling. Walking on slopes introduces additional complexities and demands on the body, leading to altered gait biomechanics, including a range of adaptations to spatiotemporal parameters^1,2^, kinematic patterns^2–7^, joint kinetics^2,5, 6,8,9^, and muscle forces^10–15^, which impact stability during gait^16–18^. Walking on slopes also poses an increased risk of slip-/trip-related falls compared to level walking due to the increased shear component of ground reaction force (GRF)^19,20^; however, the mechanisms of balance recovery during sloped walking are largely unknown.

Nonetheless, numerous recent studies of level walking have described the biomechanical features of balance recovery after disturbances in both overground and treadmill frameworks^21,22^. For example, in simulated slip perturbations, individuals demonstrated a complex sequence of counter-balancing and correction involving modulation of foot placement and segmental angular momentum in both sagittal and frontal planes during subsequent recovery^23,24^. Recovery from trip perturbations showed greater complexity, with the timing of the trip and the framework, overground or treadmill, crucial in determining which limb is the recovery limb and the specific response strategy^21^. Despite this, slips are reportedly more frequent in occupational settings^25^ and are more likely to result in fractures than trips^26^. Given the further elevated risks of slip-/trip-related falls on slopes, understanding balance recovery in these specific scenarios may benefit clinical interventions such as perturbation-based balance training^22,27^ for populations with impaired balance.

Two metrics, whole-body angular momentum (WBAM)^28^ and margin of stability (MoS)^29^ have been used to quantify stability during gait. WBAM quantifies the rotational momentum of the entire body around the center of mass (CoM)^28^, while MoS is related to the impulse required to induce instability and is defined as the distance between the extrapolated trajectory of the body’s centre of mass and the boundaries of its base of support (BoS)^29,30^. MoS has been used to quantify perturbation response (PR), which describes an individual’s capacity to recover from a perturbation during gait within a finite number of steps^31^. During walking, regulating WBAM and MoS is crucial for maintaining dynamic balance^28–30,32–35^. Poor control of WBAM and MoS are correlated with compromised balance^36^ and elevated fall risk^37,38^.

Studies of WBAM and MoS during sloped and perturbed walking are few but have provided considerable insight into gait stability. Specifically, WBAM is more tightly regulated when walking downhill, where the risk of falling is potentially higher compared to uphill walking^33,39^. With respect to perturbations, WBAM has been studied in level walking following overground trips^34,40^, multidirectional slip-like, and platform translation perturbations^41,42^. Leestma et al.^42^ noted a strong correlation between frontal plane integrated WBAM and step width and a somewhat weaker correlation between sagittal plane integrated WBAM and step length during perturbed steps. Furthermore, WBAM has been utilized in other investigations to explore factors such as the impact of anticipation^35^ and slow walking^43^ on recovery. Thus, although the effects of slope and perturbation type (slip or trip) on gait have been studied independently, to the best of our knowledge, no studies to date have examined their combined effects on walking stability.

Therefore, this study aimed to investigate the effects of slips and trips on balance recovery during level and sloped walking using a treadmill-based framework. Our objectives were: (1) to quantify WBAM and PR during walking with treadmill-induced belt acceleration (slip) and deceleration (trip) perturbations, respectively, and (2) to compare the effects of these simulated hazards during level, uphill, and downhill walking on balance recovery. Although trip recovery is a more complex process, given that slipping confers greater fall risk than tripping^26^, we hypothesized that a slip perturbation during gait would more substantially increase WBAM and PR compared to the trip perturbation and, therefore, pose a greater challenge for recovery in all three walking scenarios. We further hypothesized that these perturbations during sloped walking would elicit greater changes in our stability measures compared to level walking, with downhill walking demonstrating the greatest decrease in MoS, increase in WBAM, and increase in PR, posing the greatest challenge for recovery.

## Methods

### Participant

Eighteen healthy young adults (9 male and 9 females; age: 24.7 ± 2.5 years; height: 1.71 ± 0.09 m; mass: 70.1 ± 9.8 kg) were recruited. Participants met inclusion criteria of no prior lower extremity injuries, diseases, surgeries, or balance-compromising medications. Ethical approval was granted by the Human Research Ethics Committee (HREC) of the University of Melbourne (Ethics ID: 1954908.1). All participants provided written informed consent to participate in the study, and all experiments were carried out in accordance with the HREC-approved guidelines, which includes the Australian National Statement of Ethical Conduct in Human Research.

### Test protocol

The experiment was conducted at the Computer Assisted Rehabilitation ENvironment (CAREN, Motek Medical B.V, Netherlands) laboratory at the University of Melbourne. CAREN features a 10-camera motion capture system (Vicon Inc; Oxford, United Kingdom), recording data at 100 Hz, and a 1 × 2 m split-belt treadmill instrumented with two force plates, sampled at 1000 Hz (Motekforce Link, The Netherlands). For each participant, twenty-six infrared-reflective markers were positioned on the bony landmarks of the pelvis, trunk, and each foot, lower leg, and thigh, using a modified Helen Hayes protocol^44^. During each experimental trial, the three-dimensional trajectories of these markers were recorded using the motion capture system, and ground reaction forces (GRF) on each leg were simultaneously recorded using the force plates. The recorded GRF and marker trajectories were low-pass filtered using a second-order Butterworth filter with cut-off frequencies of 10 Hz^45^. Initially, each participant undertook a static standing calibration trial with arms held in a T-configuration. Subsequently, the participant walked barefoot on the split-belt treadmill for approximately two minutes at 1.25 m/s to familiarize themselves with the treadmill and environment at zero gradient (i.e., level walking). The participant then undertook walking trials at 1.25 m/s on the treadmill at three different surface gradients: − 8°, 0, and + 8°. At each slope setting, the participant walked for 1 min as the baseline measurement, followed by two unanticipated trip and slip perturbations administered to the right limb with at least a 20 s washout period between each perturbation. Trip-like perturbations were administered by a rapid deceleration of the right belt to 0.4 m/s with a maximum acceleration of 5 m/s^2^ followed by a rapid acceleration to the baseline speed. Additionally, slip-like perturbations were induced by accelerating the belt to 2.1 m/s followed by a swift deceleration to the baseline speed^46^. The protocol was designed and executed using the D-Flow software (Motek Medical B.V, Houten, The Netherlands), with perturbations triggered immediately after the right foot's heel strike. To avoid any potential learning bias, each participant's order of the perturbations and slope conditions was randomized.

### Biomechanical modelling

Biomechanical modelling was undertaken using OpenSim 4.1^47^. For each participant, the static calibration trial was used to scale a generic 23-degree-of-freedom musculoskeletal model (gait2392) to that participant’s anthropometry. For each recorded trial for that participant, the time history of joint angles was calculated using an inverse kinematics approach, which found the model pose at each time step that minimized the global sum of squared errors between each experimentally recorded marker and its equivalent virtual marker on the model. Subsequently, the corresponding kinematics (i.e., linear and angular positions, velocities, and accelerations) of each individual body segment and the whole-body CoM (hereafter referred to as the CoM) were calculated.

### Measures of stability

#### Margin of stability (MoS)

For each recorded trial, to determine the MoS, we first calculated *XCoM*, the instantaneous “extrapolated CoM” in three dimensions as defined by Hof et al., at each time step based on the instantaneous position and linear velocity of the CoM. For treadmill gait, at each time step, this is given by the vector expression:

1 $$XCoM={\mathbf{r}}_{CoM}+ \frac{{\mathbf{v}}_{CoM}+|{\mathbf{v}}_{belt}|}{{\omega }_{0}}$$

where ${\mathbf{r}}_{CoM}$ is the instantaneous position of the CoM, ${\mathbf{v}}_{CoM}$ is the instantaneous velocity vector of the CoM, and ${\omega }_{0}=\sqrt{g/l}$ is the eigenfrequency of the whole body modelled as a single inverted pendulum with *l* as the distance of body CoM to the lateral malleolus marker at heel strike and gravitational acceleration *g*. The treadmill velocity is represented as $${\mathbf{v}}_{belt}=\left[\begin{array}{ccc}-vcos(\theta )& 0& 0\end{array}\right]$$ where *v* m/s is the belt speed.

**Figure 1:**
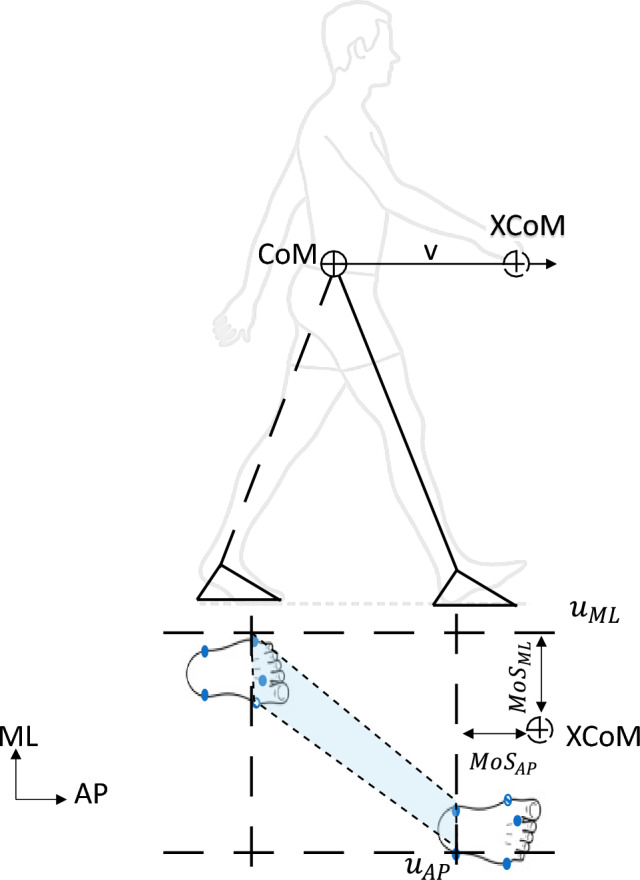
Illustrating margin of stability (MoS) calculation in anteroposterior (AP) and mediolateral (ML) directions. The shaded area represents the base of support (BoS).

At each time step, the MoS was then calculated as the minimum Euclidean distance between XCoM and the boundaries of the base of support (BoS), defined as the convex hull enclosing the stance foot or feet in single- or double-support respectively (Fig. 1). The BoS was estimated using the spatial position of markers attached to the participant’s feet. For the purposes of this study, we calculated separate anteroposterior (AP) and mediolateral (ML) margins of stability using the AP and ML components of the XcoM, respectively:

2 $$Mo{S}_{AP}={u}_{AP}-{XCoM}_{AP}, \, Mo{S}_{ML}={u}_{ML}-{XCoM}_{ML}$$

where ${u}_{AP}$ and ${u}_{ML}$ are the locations of the edges of the BoS nearest to the XCoM along the AP and ML directions, respectively (Fig. 1)^48,49^. In each direction, positive MoS indicates that XCoM is within the BoS and is, therefore, a stable configuration, while negative MoS indicates instability and inevitable fall without: (1) a change in the base of support to counter this action, such as a corrective step; (2) the use of inertial strategies to arrest any potential fall, such as rapid neuromuscular modulation of skeletal configuration and ground forces; or (3) a combination of both..

#### Perturbation response (PR)

To quantify the destabilizing effects of perturbations on stability, the perturbation effect was quantified using the perturbation response (PR) measure, a measure derived from the gait sensitivity norm^50^. Any indicative quantitative measure of perturbed gait can be used in the PR formulation. For our present study, we applied the MoS as the indicative measure to calculate the PR along the AP and ML coordinates for each perturbation type (slip or trip):

3 $$PR_{AP} = \sqrt {\mathop \sum \limits_{i = 1}^{q} \left( {\frac{{MoS_{AP}^{i} - \overline{MoS}_{AP}^{*} }}{{\sigma_{AP}^{*} }}} \right)^{2} } ,{ }PR_{ML} = \sqrt {\mathop \sum \limits_{i = 1}^{q} \left( {\frac{{MoS_{ML}^{i} - \overline{MoS}_{ML}^{*} }}{{\sigma_{ML}^{*} }}} \right)^{2} ,}$$

where ${\overline{MoS} }^{*}$ and ${\sigma }^{*}$ are the mean and standard deviation, respectively, of the instantaneous MoS at heel strike of 20 unperturbed gait cycles, ${MOS}^{i}$ is the instantaneous MoS at heel strike for the *i*th step after the perturbation was applied. AP and ML are the anteroposterior and mediolateral components, respectively. For the present study, we set the total number of steps after perturbation $q=6$ consistent with previous research^31^, as it was observed that participants fully regained their normal gait patterns after six steps. Smaller values of PR indicate less deviation from unperturbed walking.

#### Whole-body angular momentum (WBAM)

For each recorded trial, the three-dimensional WBAM was calculated with respect to the whole-body center of mass as the sum of individual angular momenta of each body segment normalized to the participant’s mass *M*, walking velocity *V*, and height *H*:

4 $$WBAM=\frac{\sum_{i=1}^{n}\left[\left({\mathbf{r}}_{CoM}^{i}-{\mathbf{r}}_{CoM}\right)\times {m}_{i}\left({\mathbf{v}}_{CoM}^{i}-{\mathbf{v}}_{CoM}\right)+{\mathbf{I}}^{i}{{\varvec{\upomega}}}^{i}\right]}{MVH}$$

where ${\mathbf{r}}_{CoM}$ and ${\mathbf{v}}_{CoM}$ are the instantaneous position and linear velocity vectors of the CoM respectively, ${\mathbf{r}}_{CoM}^{i}$ and ${\mathbf{v}}_{CoM}^{i}$ are the instantaneous position and linear velocity vectors of the centre of mass of the *i*th body segment respectively, ${\mathbf{I}}^{i}$ and ${{\varvec{\upomega}}}^{i}$ are the moment of inertia and the angular velocity about the segment’s own centre of mass for the *i*th segment represented in the world coordinate system, respectively. The integrated WBAM (iWBAM) was also calculated by integrating the WBAM waveform over time. For the purposes of this study, only the sagittal- and frontal-plane components of WBAM and iWBAM were considered.

### Statistical analysis

In order to investigate our stated hypotheses, we focused on one temporal measure of stability: WBAM waveform; and three discrete measures of stability: PR (using MoS), iWBAM; and the peak-to-peak WBAM range. In addition to these hypothesis-driven tests, we conducted exploratory analyses to provide a comprehensive understanding of the recovery strategies involving two additional discrete variables: step length and step width. For each participant, primary outcome measures were calculated for each slope condition (− 8°, 0, + 8°) and perturbation direction (slip or trip) using all recorded trials for that participant. Discrete variables were subsequently analyzed in SPSS (IBM Corp., NY, USA) using a two-way repeated measures ANOVA to study the effects of both perturbation direction and slopes as well as their interaction. In case of observed significant differences, post-hoc pairwise t-tests with Bonferroni corrections were performed. For each perturbation direction and slope condition, WBAM was analysed using a one-way repeated measures ANOVA with statistical parametric mapping (SPM)^51^ (www.spm1d.org) in MATLAB (R2021a, The MathWorks Inc). The slope was set as the independent variable. Where significant differences between slope conditions were found, post-hoc SPM t-tests with Bonferroni corrections were performed to determine which pair of slope conditions differed significantly. For all statistical analyses, significance was set at $\mathrm{\alpha }<0.05$.

## Results

Due to the large volume of results from one- and two-way ANOVAs with post-hoc analyses, only significant results and associated p-values are discussed briefly below. Complete results of statistical analyses, with all p-values, are provided as Supplementary Material.

### Perturbation response

**Figure 2:**
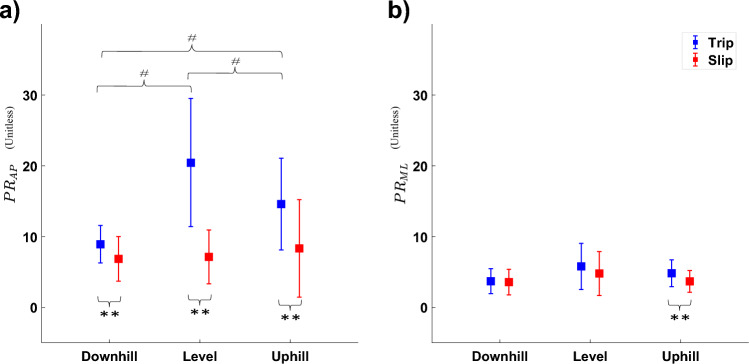
Perturbation response (PR) (**a**) in the anteroposterior (PR_AP_); and (**b**) in the mediolateral (PR_ML_) directions during level, downhill, and uphill walking conditions in response to slip and trip perturbations. *Depicts significant difference compared to Unperturbed. **Depicts significant difference between trip and slip recovery. #Depicts significant effects of slope.

Two-way ANOVA (Table 1 and Fig. 2a) found significant differences in anteroposterior PR between slopes, perturbation type and their interaction, with subsequent post-hoc testing showing a significant differences between uphill and level (mean diff: 5.89; 95% CI [1.022;10.754]; *p* = 0.021), downhill and level (mean diff: 11.556; 95% CI [5.615;17.498]; *p* < 0.001), and uphill and downhill slopes for trip (mean diff: 5.67; 95% CI [1.716,9.62]; *p* = 0.008). Post-hoc testing also showed significant differences between slip and trip perturbation across all slopes (slip vs. trip: Level: mean diff: − 13.287; 95% CI − 17.764; − 8.813]; *p* < 0.001; uphill: mean diff: − 6.277; 95% CI [− 9.785; − 2.759]; *p* = 0.002; downhill: mean diff: − 2.062; 95% CI [− 3.745; − 0.379]; *p* = 0.019) (Supplementary material Tables S3 and S4). Two-way ANOVA (Table 1 and Fig. 2b) found significant differences in mediolateral PR between slopes, with subsequent post-hoc testing showing a significant difference between slip and trip PR only during uphill walking (mean diff: − 1.121; 95% CI [− 1.840; − 0.401]; *p* = 0.004) (Supplementary material Tables S5 and S6). No other significant differences were found.

**Table 1:** Two-way ANOVA results with slope and perturbation type as independent factors.

		Two-way ANOVA results											
		Perturbation				Slope				Perturbation × slope			
Ranges		df	F	Sig	${\eta }_{p}^{2}$	df	F	Sig	${\eta }_{p}^{2}$	df	F	Sig	${\eta }_{p}^{2}$
PR	MoS_AP	1	52.798	< .001	.756	2	6.577	.004	.279	2	12.998	< .001	.433
	MoS_ML	1	6.236	.023	.268	2	3.589	.039	.174	2	1.211	.310	.067
Range	Sagittal (Z)	2	23.372	< .001	.579	2	31.752	< .001	.651	4	2.342	.064	.121
	Frontal (X)	2	9.225	< .001	.352	2	40.345	< .001	.704	4	0.347	.845	.02
iWBAM	Sagittal (Z)	2	28.082	< .001	.623	2	46.913	< .001	.734	4	7.800	< .001	.315
	Frontal (X)	2	13.999	< .001	.452	2	15.593	< .001	.478	4	1.505	.21	.081
Step length		2	58.951	< .001	.776	2	10.965	< .001	.392	4	2.326	.065	.120
Step width		2	36.576	< .001	.683	2	.713	.497	.040	4	2.586	.044	.132

Perturbation types: slip, trip, and unperturbed.

Slopes: downhill (− 8°); level (0°) and uphill (+ 8°).

Statistical significance was set at $\alpha < 0.05$ and effect sizes are shown as partial eta square (${\eta }_{p}^{2}$).

### Spatiotemporal adjustments

Two-way ANOVA (Tables 1, 2) found significant differences in step length between slopes, perturbation types, and their interactions. Step width was only significantly different for perturbation type, but not slopes and their interaction. Post-hoc tests on the main effects found significant differences between pairs of conditions as described below and shown in Table 2. Full ANOVA results are provided as Supplementary Material.

**Table 2:** Mean and standard deviation of spatiotemporal step measures normalized by the leg length (LL) during level and sloped walking with and without perturbation.

	Downhill walking			Level walking			Uphill walking		
	Unperturbed	Trip	Slip	Unperturbed	Trip	Slip	Unperturbed	Trip	Slip
Step length (/LL)	0.623^#^ (0.059)	0.441*^,^** (0.13)	0.551*^,^** (0.096)	0.704 (0.041)	0.445*^,^** (0.099)	0.6549** (0.047)	0.703 (0.085)	0.537*^,^** (0.15)	0.654** (0.11)
Step width (/LL)	0.197 (0.045)	0.234* (0.057)	0.222 (0.048)	0.169 (0.039)	0.237* (0.048)	0.241* (0.066)	0.187^#^ (0.015)	0.246* (0.055)	0.244* (0.052)

Results presented as: mean (standard deviation).

Significant differences were determined using post-hoc pairwise *t*-tests after two-way ANOVA (Table 1).

Statistical significance with Bonferroni correction was set at $\alpha < 0.017$.

*Significant difference compared to unperturbed.

**Significant difference between trip and slip.

^#^Significant effect of slope.

#### Effect of slope

Across all tested perturbation types (slip, trip, and unperturbed), downhill walking resulted in smaller step lengths compared to level walking (Table 2); however, significance was reached only in the unperturbed condition (downhill: *p* < 0.001). Compared to level walking, larger step widths were found for sloped walking across all perturbation types (Table 2), uphill: *p* < 0.001; downhill: *p* < 0.001). No other significant differences in step length or width due to the slope main effect were found.

#### Effect of perturbation

During sloped walking, step length decreased significantly during slip (downhill: *p* < 0.001) and trip (uphill: *p* < 0.001; downhill: *p* < 0.001) perturbations compared to the unperturbed condition. Furthermore, the trip condition produced significantly smaller step lengths compared to the slip condition (uphill: *p* = 0.038; downhill: *p* = 0.014). For level walking, both perturbation types reduced step lengths, with trips producing greater change than slips (*p* < 0.001, Table 2). Step width increased significantly during slip in level and uphill walking (uphill: *p* = 0.002; level: *p* < 0.001) and trip (uphill: *p* = 0.001; downhill: *p* = 0.008; level: *p* < 0.001) perturbations compared to the unperturbed condition (Table 2).

### WBAM

#### Effect of slope on unperturbed walking

**Figure 3:**
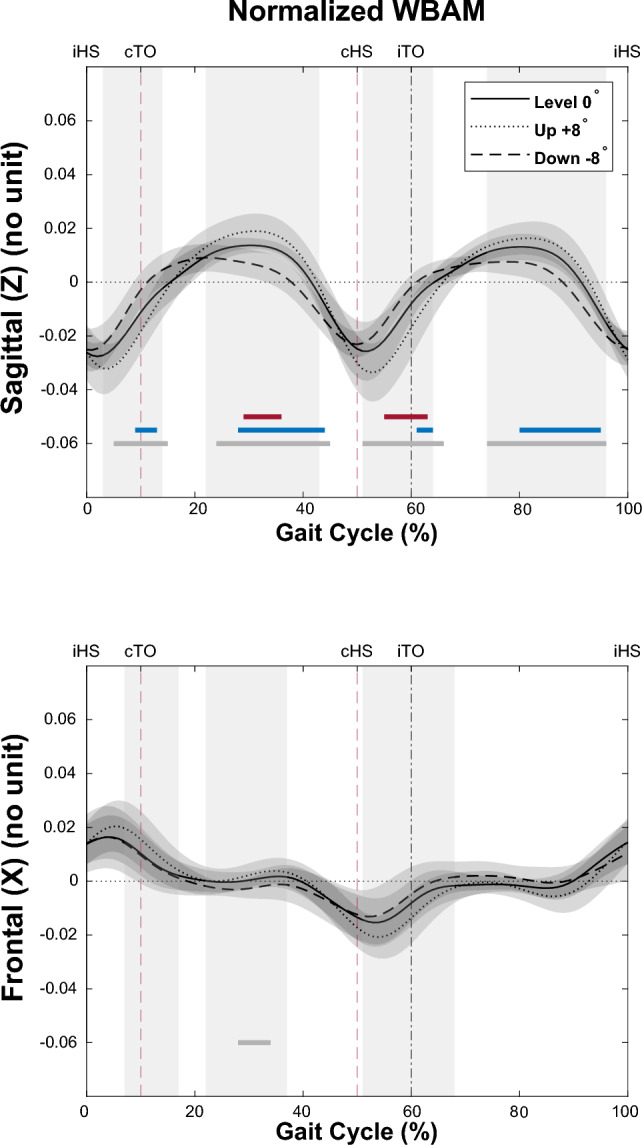
Whole-body angular momentum (WBAM) in the Sagittal (Z) and Frontal (X) planes during unperturbed Level (solid black line), Downhill (dashed black line), and Uphill (dotted black line) walking. The vertical shaded areas indicate significant differences between waveform data of Level, Downhill, and Uphill, as determined by a one-way repeated measures ANOVA SPM. Below the curves, the horizontal lines represent post-hoc results with corrected $\mathrm{\alpha }$ <0.0167, where the blue bar indicates a significant difference observed in pairwise comparison between level and downhill, the red bar indicates a significant difference between level and uphill, and the gray lines indicate significant differences between uphill and downhill. The vertical dashed lines denote gait events namely: ipsilateral heel strike (iHS), contralateral toe off (cTO), contralateral heel strike (cHS), ipsilateral toe off (iTO). More detailed results of the SPM ANOVA and post-hoc tests can be found in the supplementary material Table S1 and Table S2.

One-way repeated measures ANOVA using SPM found significant differences in WBAM due to slope (uphill, downhill, and level) throughout most of the gait cycle in both the sagittal and frontal planes (Fig. 3, vertical shaded areas). Furthermore, post-hoc tests for the sagittal plane found regions of statistical significance throughout the gait cycle for all three pairs of comparisons. In the frontal plane, regions of significant difference between uphill and downhill walking were found during mid-stance and only (Fig. 3). Full results of SPM ANOVA and post-hoc tests are provided as Supplementary Material (Tables S1 and S2).

#### Effect of perturbation

**Figure 4:**
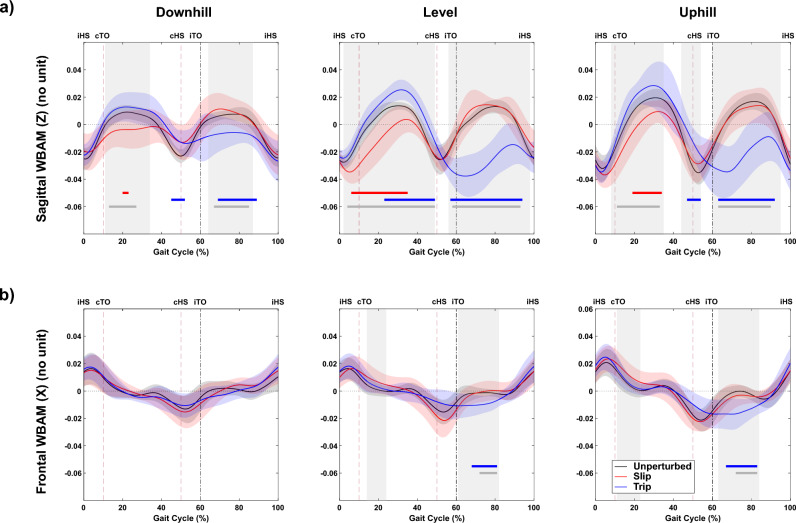
Whole-body angular momentum (WBAM) in (**a**) sagittal and (**b**) frontal planes during unperturbed (solid black curve), perturbed slip (red curve), and perturbed trip (blue curve) conditions. The shaded vertical areas indicate significant differences between waveform data of Unperturbed, Slip, and Trip Perturbations, as determined by a one-way repeated measures ANOVA (SPM results) for downhill, level, and uphill walking with perturbation type as the independent variable. Below the curves, horizontal lines represent post-hoc results with corrected $\mathrm{\alpha }$ <0.0167. The blue bar indicates a significant difference between unperturbed and trip conditions, the red bar signifies a significant difference between unperturbed and slip conditions, and gray lines represent significant differences between Slip and Trip perturbations. Vertical dashed lines denote gait events, specifically: ipsilateral heel strike (iHS), contralateral toe off (cTO), contralateral heel strike (cHS), and ipsilateral toe off (iTO).

**Figure 5:**
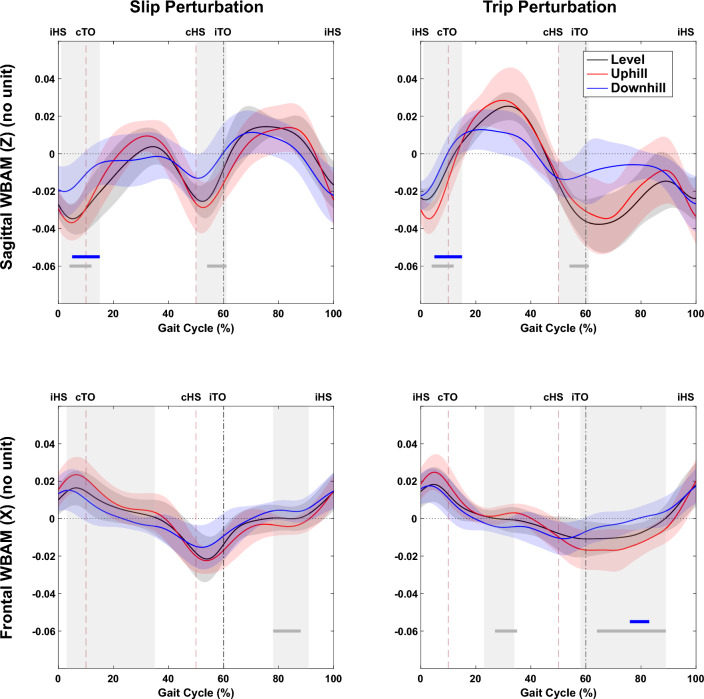
Whole-body angular momentum (WBAM) in sagittal and frontal planes during perturbed level walking (solid black curve), perturbed uphill (red curve), and perturbed downhill (blue curve) during slip and trip perturbations. The vertical shaded areas indicate significant differences between waveform data of level, uphill and downhill curved during perturbations as determined by a one-way repeated measures ANOVA (SPM results) for both slip and trip perturbations with slope as the independent variable. The horizontal lines below the curves represent post-hoc results with corrected $\mathrm{\alpha }$ < 0.0167, where the blue bar indicates a significant difference between level and downhill, the red bar indicates a significant difference between level and uphill, and the gray lines indicate significant differences between uphill and downhill perturbed walking. The vertical dashed lines denote gait events namely: ipsilateral heel strike (iHS), contralateral toe off (cTO), contralateral heel strike (cHS), ipsilateral toe off (iTO).

Across all slopes (uphill, downhill, and level walking), one-way repeated measures ANOVA using SPM found significant differences in sagittal-plane WBAM due to perturbation type occurring throughout mid-stance and most of the swing phase (Fig. 4a). Post-hoc tests found trip perturbations to be associated with, statistically significant, differences in sagittal-plane WBAM during the swing phase, across all slopes. In contrast, slip perturbations were associated with significant differences in sagittal-plane WBAM through early to mid-stance, that were statistically significantly different only for level and uphill walking (Fig. 4a). In the frontal plane, significant differences in WBAM were found only during mid-swing for level and uphill walking (Fig. 4b). Post-hoc tests found these to result from statistically significant, differences associated with trip perturbations. Figure 5 shows the result of one-way repeated measures ANOVA using SPM, with slope as independent variable for both trip and slip perturbations. Despite significant differences in WBAM curves between the three slopes during unperturbed walking (Fig. 4), no significant differences were detected after perturbation.

### iWBAM and range of WBAM

In both frontal and sagittal planes, two-way ANOVA (Table 1) found significant differences in range of WBAM between slopes (*p* < 0.001) and perturbation types (*p* < 0.001) and but not their interaction. Two-way ANOVA results for iWBAM in the sagittal and frontal planes largely reflected those of the range of WBAM. Significant differences in iWBAM were found between slopes (*p* < 0.001) and perturbation types (*p* < 0.001), with a significant interaction found for the sagittal plane only (*p* < 0.001). Post-hoc tests on the main effects found significant differences between pairs of conditions as shown in Table 3.

**Table 3:** Mean and standard deviation of WBAM range and integral (iWBAM) in sagittal and frontal planes during level and sloped walking with and without perturbation.

		Downhill walking			Level walking			Uphill walking		
		Unperturbed	Trip	Slip	Unperturbed	Trip	Slip	Unperturbed	Trip	Slip
WBAM range	Sagittal (Z)	0.043 (0.008)	0.0674* (0.024)	0.051 (0.013)	0.0476 (0.009)	0.068* (0.024)	0.059* (0.015)	0.0627^#^ (0.015)	0.837*^,^** (0.017)	0.066** (0.017)
	Frontal (X)	0.0334 (0.019)	0.0357 (0.017)	0.0382 (0.019)	0.034 (0.015)	0.0379 (0.017)	0.043 * (0.018)	0.045^#^ (0.017)	0.0496 (0.018)	0.0515 (0.015)
iWBAM	Sagittal (Z)	1.128^#^ (0.193)	1.3412 (0.401)	1.383 * (0.339)	1.336 (0.229)	2.23 *^,^** (0.802)	1.68*^,^** (0.451)	1.804^#^ (0.332)	2.467 *^,^** (0.514)	1.794** (0.471)
	Frontal (X)	0.762 (0.415)	0.831 (0.463)	0.906 (0.504)	0.727 (0.351)	0.912 ** (0.463)	1.05 *^,^** (0.517)	0.953^#^ (0.478)	1.231* (0.494)	1.208* (0.452)

Results presented as: mean (standard deviation).

Significant differences were determined using post-hoc pairwise *t*-tests after two-way ANOVA (Table 1).

Statistical significance with Bonferroni correction was set at $\alpha < 0.017$.

*Significant Difference compared to Unperturbed.

**Significant Difference between Trip and Slip.

^#^Significant effects of slope.

#### Effect of slope

Regardless of perturbation type, both sagittal-plane iWBAM and range of WBAM increased with increasing slope, from downhill to level to uphill (Table 3). Post-hoc tests found statistically significant differences for all three comparison pairs in the unperturbed condition only, for sagittal plane iWBAM (uphill-downhill: *p* < 0.001; uphill-level: *p* < 0.001; downhill-level: *p* = 0.012, Table 3) and range of WBAM (uphill–downhill: *p* < 0.001; uphill-level: *p* < 0.001; (Table 3). Similarly, both the frontal-plane iWBAM and range of WBAM increased with uphill walking (Table 3). However, significant differences were only found between unperturbed uphill and level walking (iWBAM: *p* < 0.001; range of WBAM: *p* < 0.001). Additionally, a significant difference was found between unperturbed downhill and uphill walking for the range of WBAM (*p* = 0.007) in the frontal plane.

#### Effect of perturbation

Regardless of slope, both sagittal plane iWBAM and range of WBAM were always greatest for trip perturbations and smallest in the unperturbed condition, with slip perturbations intermediate (Table 3). Post-hoc tests for sagittal plane iWBAM and range of WBAM found that perturbed walking was always significantly different to the unperturbed condition regardless of slope, except for trip perturbations during downhill walking (Table 3). Furthermore, significant differences were found between slip and trip perturbations for uphill (iWBAM: *p* < 0.001; range of WBAM: *p* < 0.001) and level (iWBAM: *p* = 0.025) walking. In contrast, frontal-plane iWBAM and range of WBAM was always greatest for slip perturbations and smallest for unperturbed walking, with trip perturbations intermediate (Table 3). A number of significant pairwise differences between perturbed walking and the unperturbed condition at various slopes were found for frontal plane iWBAM and range of WBAM (Table 3).

## Discussion

The primary goal of this study was to investigate the effects of slips and trips, common and hazardous disturbances encountered during gait, on balance recovery during both level and sloped walking using treadmill-induced perturbations. We aimed to quantify MoS, WBAM, and PR in response to these perturbations and to compare the impacts across different surface inclinations.

Contrary to our first hypotheses, slip perturbations did not consistently induce greater alterations in WBAM and PR compared to trip perturbations. Specifically, trip perturbations had more destabilizing effects than slip perturbations in level walking and both inclined and declined walking, as indicated by the larger PR and greater changes in step length and width in all conditions. Our findings for sloped walking thus agree with and extend those of a study of PR during perturbed level walking by Roeles et al.^31^. Furthermore, although slip and trip perturbations produced opposite effects on WBAM during walking, the larger destabilization introduced by trips is clearly evident in the greater deviation of WBAM relative to unperturbed walking across all slopes. This is especially so during the swing phase of the perturbed limb during the recovery step. Thus, our finding that trip-like perturbations may induce larger destabilizing effects during sloped walking also lends greater weight to a growing body of research using treadmill and overground frameworks, suggesting recovery from trips may be more difficult and complex than slips^52^.

However, some contrasting findings have been reported. Ren et al.^53^ demonstrated that recovery from a slip-like perturbation required greater hip extension, hip adduction, and knee extension moments compared to a trip. This led the authors to conclude that recovery from slipping was more challenging than recovery from tripping^53^. Additionally, Lee et al.^54^ reported that trunk and whole body center of mass movements, along with increased muscle activities in the tibialis anterior, gastrocnemius, rectus femoris, and biceps femoris, were higher following slip-induced perturbations compared to trip-induced perturbations, further suggesting that slipping had greater effects. These discrepancies can be attributed to differences in the perturbation protocols and intensities employed across studies. Factors such as the duration, magnitude^55^, and timing^56^ of the perturbations can significantly influence the observed effects, leading to differing conclusions.

Surprisingly, our observations revealed that PR was higher during level walking compared to both the downhill and uphill walking conditions, regardless of perturbation type. This unexpected finding contradicts our initial hypothesis, which proposed that recovering from perturbations would be more challenging during sloped walking. The higher PR observed during level walking suggests that individuals experienced more substantial deviations in MoS and faced increased challenges to maintaining stability compared to walking on inclined surfaces. Although we did not specifically test psychological factors in our present study, a possible explanation for our finding is that individuals may have more confidence and less fear of falling when walking on a level surface. In contrast, during downhill walking, individuals adopt a more conservative gait pattern^13^ and are more vigilant due to the fear of falling, hence, faster and more adaptive reactions to prevent potential falls^12,13,19^. Similarly, during uphill walking, the higher mechanical demands placed on the body may prompt individuals to be more attentive and responsive to perturbations. Nevertheless, our findings reinforce the robustness of human balance recovery strategies over varied terrain. Regardless of slope or perturbation type, the response was similar: reduced step length and increased step width.

Our findings revealed that PR in the anteroposterior direction consistently exceeded PR in the mediolateral direction, regardless of perturbation type and surface inclination. Thus, participants recovered their mediolateral stability more quickly than their anteroposterior stability, in agreement with a treadmill-based perturbation study by Madehkhaksar et al.^57^. Stability was recovered rapidly in the mediolateral direction simply by widening the base of support through increased step width, i.e., greater lateral foot placement relative to the upper extremity as described by Rasmussen and Hunt^23^. In the anteroposterior direction, however, the application of a concomitant anteroposterior perturbation created a more complex scenario in which the central nervous system needed to act to recover from the large fore-aft perturbation without falling while simultaneously maintaining the set treadmill speed. Naturally, this more complex response may have brought the larger PR in the anteroposterior direction. Thus, step length adjustment, which upon application of the perturbation was initially shortened, then steadily returned to normal, required concomitant modulation of step frequency to maintain the set treadmill speed. This was especially so during sloped walking, in which step length was shortened up to 38% in the case of a trip perturbation during uphill walking (Table 2). However, walking speed may also be modulated during overground walking to help maintain stability; therefore, our present findings for treadmill walking may not apply to overground walking. This may be especially so for trip perturbations, where several recent studies have found considerable differences in recovery mechanics between treadmill and overground frameworks^21,58^.

Our study has certain limitations that need to be acknowledged. Firstly, we used only two surface inclinations, ± 8°^11^. These inclinations were chosen based on participant comfort and comparability with existing literature. However, this limited range of surface inclinations restricts the generalizability of our results to a broader range of slope angles; therefore, our findings may differ during perturbed walking on very steep slope angles. Our results, in accordance with previous studies^33,39^, showed that WBAM in the sagittal plane is regulated more strictly during downhill walking than level and uphill walking at 8°. This tighter regulation of WBAM is likely a strategy to maintain balance and prevent excessive forward momentum generated by the downhill slope^33^. However, Silverman et al.^33^ showed that the range of sagittal-plane WBAM is lowest at about 5° of decline, increasing gradually but significantly with greater decline angle. Conversely, the range of sagittal-plane WBAM almost doubles between level walking and 15° of incline. These findings demonstrate that the tight regulation of WBAM becomes substantially more difficult at high inclines and declines. Thus, it is possible that the associated perturbation recovery strategies may differ from our present findings due to the concomitant challenge of maintaining upright gait on such steep slopes.

Secondly, we induced perturbations using a split-belt treadmill at the instant of heel strike, which we again acknowledge may not completely reflect the effects of over-ground slips or trips^21^. Although overground walking may better reflect real-world slip/trip scenarios, a treadmill protocol permitted a high level of control and repeatability with respect to the perturbation characteristics, including perturbation intensity, randomization, and the onset timing during the gait cycle. With respect to the latter, we chose to apply the perturbation at heel strike for consistency both within our study and across published studies to date^22^. However, onset timing of perturbation during the gait cycle has been shown to be important, particularly during trips^21,56^. Therefore, future research should address the effects of different onset timings and intensities on recovery strategies.

Thirdly, we employed a fixed walking speed to ensure consistency with previous studies and minimize any potential confounding factors associated with variability in walking speed. This speed was found to be comfortable in our pilot study in all slope conditions. Nevertheless, we acknowledge that walking at participants’ individual preferred speeds would have provided results with greater participant-specificity, however, given the good agreement between our results and those of previous studies of both sloped walking^33^ and perturbed walking, we are confident that our broader findings would not have been affected.

Finally, our findings are specific to young, healthy adults and may not fully generalize to older adults or other populations at greater risk of falls. Our findings support the notion that recovery from trips is a more complex process, thus future research is needed to resolve the underlying reasons why slips, rather than, trips more often result in falls and subsequent injury, which will have important implications for falls prevention in these high-risk populations.

### Supplementary Information

Supplementary Information 1.

Supplementary Tables.

## Data Availability

The datasets generated during and/or analysed during the current study are included in this published article.

## References

[1] Kawamura K, Tokuhiro A, Takechi H (1991). Gait analysis of slope walking: A study on step length, stride width, time factors and deviation in the center of pressure. Acta Med. Okayama.

[2] Strutzenberger G (2022). Gait on slopes: Differences in temporo–spatial, kinematic and kinetic gait parameters between walking on a ramp and on a treadmill. Gait Posture.

[3] Sarvestan J (2021). Lower limb joint angles and their variability during uphill walking. Gait Posture.

[4] Simpson K (1993). Kinematic and plantar pressure adjustments to downhill gradients during gait. Gait Posture.

[5] Kuster M, Sakurai S, Wood GA (1995). Kinematic and kinetic comparison of downhill and level walking. Clin. Biomech..

[6] Lay AN, Hass CJ, Gregor RJ (2006). The effects of sloped surfaces on locomotion: A kinematic and kinetic analysis. J. Biomech..

[7] Zeng X (2022). The 6DOF knee kinematics of healthy subjects during sloped walking compared to level walking. Gait Posture.

[8] Haggerty M (2014). The influence of incline walking on joint mechanics. Gait Posture.

[9] Pacifico D (2020). Discriminant validity and reproducibility of spatiotemporal and kinetic parameters during treadmill walking in patients with knee osteoarthritis. Gait Posture.

[10] Franz JR, Kram R (2012). The effects of grade and speed on leg muscle activations during walking. Gait Posture.

[11] Pickle NT (2016). The functional roles of muscles during sloped walking. J. Biomech..

[12] Franz JR, Kram R (2013). How does age affect leg muscle activity/coactivity during uphill and downhill walking?. Gait Posture.

[13] Dewolf AH, Mesquita RM, Willems PA (2020). Intra-limb and muscular coordination during walking on slopes. Eur. J. Appl. Physiol..

[14] Alexander N (2021). Effect of different walking speeds on joint and muscle force estimation using AnyBody and OpenSim. Gait Posture.

[15] Alexander N, Schwameder H (2023). A forefoot strike pattern during 18° uphill walking leads to greater ankle joint and plantar flexor loading. Gait Posture.

[16] Monsch ED, Franz CO, Dean JC (2012). The effects of gait strategy on metabolic rate and indicators of stability during downhill walking. J. Biomech..

[17] Vieira MF (2017). Gait stability, variability and complexity on inclined surfaces. J. Biomech..

[18] Lu, C., Al-Juaid, R., & Al-Amri, M. Gait stability characteristics in able-bodied individuals during self-paced inclined treadmill walking. https://assets.researchsquare.com/files/rs-1840726/v1/fe4e473b-b2fc-4f4b-bb18-4325e09e9a1f.pdf?c=1661767191 (2022). 10.2196/42769 PMC10280335 37276010

[19] Redfern MS (2001). Biomechanics of slips. Ergonomics.

[20] Sheehan RC, Gottschall JS (2012). At similar angles, slope walking has a greater fall risk than stair walking. Appl. Ergon..

[21] Siragy, T., Russo, Y., Young, W. & Lamb, S. E. Comparison of over-ground and treadmill perturbations for simulation of real-world slips and trips: A systematic review. *Gait Posture* **100**, 201–209 (2023). 10.1016/j.gaitpost.2022.12.015 36603326

[22] Ferreira RN (2022). Provoking artificial slips and trips towards perturbation-based balance training: A narrative review. Sensors.

[23] Rasmussen CM, Hunt NH (2021). Unconstrained slip mechanics and stepping reactions depend on slip onset timing. J. Biomech..

[24] van den Bogaart M (2020). The effect of anteroposterior perturbations on the control of the center of mass during treadmill walking. J. Biomech..

[25] Courtney TK (2001). Occupational slip, trip, and fall-related injuries can the contribution of slipperiness be isolated?. Ergonomics.

[26] Luukinen H (2000). Fracture risk associated with a fall according to type of fall among the elderly. Osteoporos. Int..

[27] McCrum C (2022). Perturbation-based balance training: Principles, mechanisms and implementation in clinical practice. Front. Sports Active Living.

[28] Herr H, Popovic M (2008). Angular momentum in human walking. J. of Exp. Biol..

[29] Hof A, Gazendam M, Sinke W (2005). The condition for dynamic stability. J. Biomech..

[30] Hof AL (2008). The ‘extrapolated center of mass’ concept suggests a simple control of balance in walking. Hum. Mov. Sci..

[31] Roeles S (2018). Gait stability in response to platform, belt, and sensory perturbations in young and older adults. Med. Biol. Eng. Comput..

[32] Neptune RR, Vistamehr A (2019). Dynamic balance during human movement: measurement and control mechanisms. J. Biomech. Eng..

[33] Silverman AK (2012). Whole-body angular momentum in incline and decline walking. J. Biomech..

[34] Pijnappels M, Bobbert MF, van Dieën JH (2004). Contribution of the support limb in control of angular momentum after tripping. J. Biomech..

[35] Eichenlaub EK (2023). Susceptibility to walking balance perturbations in young adults is largely unaffected by anticipation. Hum. Mov. Sci..

[36] Vistamehr A (2016). Correlations between measures of dynamic balance in individuals with post-stroke hemiparesis. J. Biomech..

[37] Pijnappels M, Bobbert MF, van Dieën JH (2005). Push-off reactions in recovery after tripping discriminate young subjects, older non-fallers and older fallers. Gait posture.

[38] Bierbaum S (2010). Adaptational responses in dynamic stability during disturbed walking in the elderly. J. Biomech..

[39] Pickle NT (2016). Whole-body angular momentum during sloped walking using passive and powered lower-limb prostheses. J. Biomech..

[40] Potocanac Z (2014). Fast online corrections of tripping responses. Exp. Brain Res..

[41] Martelli D (2013). Angular momentum during unexpected multidirectional perturbations delivered while walking. IEEE Trans. Biomed. Eng..

[42] Leestma JK (2023). Linking whole-body angular momentum and step placement during perturbed human walking. J. Exp. Biol..

[43] van Mierlo M (2023). Sagittal-plane balance perturbations during very slow walking: Strategies for recovering linear and angular momentum. J. Biomech..

[44] Kadaba MP, Ramakrishnan H, Wootten M (1990). Measurement of lower extremity kinematics during level walking. J. Orthop. Res..

[45] Kristianslund E, Krosshaug T, Van den Bogert AJ (2012). Effect of low pass filtering on joint moments from inverse dynamics: Implications for injury prevention. J. Biomech..

[46] Shokouhi S, Mokhtarzadeh H, Lee PV-S (2023). Lower extremity joint power and work during recovery following trip-induced perturbations. Gait Posture.

[47] Seth A (2018). OpenSim: Simulating musculoskeletal dynamics and neuromuscular control to study human and animal movement. PLoS Comput. Biol..

[48] Ohtsu H (2020). Does the balance strategy during walking in elderly persons show an association with fall risk assessment?. J. Biomech..

[49] Ohtsu H (2019). Investigation of balance strategy over gait cycle based on margin of stability. J. Biomech..

[50] Hobbelen DG, Wisse M (2007). A disturbance rejection measure for limit cycle walkers: The gait sensitivity norm. IEEE Trans. Robot..

[51] Pataky TC, Robinson MA, Vanrenterghem J (2013). Vector field statistical analysis of kinematic and force trajectories. J. Biomech..

[52] Siragy T (2020). Active arm swing and asymmetric walking leads to increased variability in trunk kinematics in young adults. J. Biomech..

[53] Ren X (2022). Lower extremity joint compensatory effects during the first recovery step following slipping and stumbling perturbations in young and older subjects. BMC Geriatr..

[54] Lee B-C, Kim C-S, Seo K-H (2019). The body’s compensatory responses to unpredictable trip and slip perturbations induced by a programmable split-belt treadmill. IEEE Trans. Neural Syst. Rehabil. Eng..

[55] Aprigliano, F., *et al*. Effects of slipping-like perturbation intensity on the dynamical stability. In *2015 37th Annual International Conference of the IEEE Engineering in Medicine and Biology Society (EMBC)*. (IEEE, 2015). 10.1109/EMBC.2015.7319586 26737486

[56] Golyski PR (2022). Onset timing of treadmill belt perturbations influences stability during walking. J. Biomech..

[57] Madehkhaksar F (2018). The effects of unexpected mechanical perturbations during treadmill walking on spatiotemporal gait parameters, and the dynamic stability measures by which to quantify postural response. PloS ONE.

[58] Rosenblatt NJ, Grabiner MD (2010). Measures of frontal plane stability during treadmill and overground walking. Gait posture.

