# Supplementary material for: Recovering whole-body angular momentum and margin of stability after treadmill-induced perturbations during sloped walking in healthy young adults
Source: Sci Rep. 2024 Feb 23;14:4421. doi: 10.1038/s41598-024-54890-4 (PMC10884438; doi:10.1038/s41598-024-54890-4)
Supplement: Supplementary file 2 — Supplementary Tables. [file 41598_2024_54890_MOESM2_ESM.docx]

Title: Recovering whole-body angular momentum and margin of stability after treadmill-induced perturbations during sloped walking in healthy young adults

Shabnam Shokouhi[a], Prasanna Sritharan[a], Peter Vee-Sin Lee[a] *

[a] Department of Biomedical Engineering, University of Melbourne, Melbourne, VIC, Australia

Authors Initials

Shokouhi Sh.[a], Sritharan P.[a], and Lee P.V.S.[a]

Corresponding address:

Prof. Peter V.S. Lee

Department of Biomedical Engineering

University of Melbourne

Victoria 3010, Australia

Email: pvlee@unimelb.edu.au

Shabnam Shokouhi (First Author)

Department of Biomedical Engineering

Email: sshokouhi@student.unimelb.edu.au

Prasanna Sritharan (Second Author)

Department of Biomedical Engineering

Email: prasanna.sritharan@unimelb.edu.au

A detailed description of the regions where a significant difference was found in post-hoc comparisons in figure 3 and figure 5 is shown in Table S1 and Table S2.

Table S1 Post-hoc Pair-wise comparison (α<0.0167) results of SPM analysis shown in Fig.3 and Fig.5 in sagittal plane (n= the number of clusters, t*= t-threshold)

Variable	*Downhill* vs *Level*				*Uphill* vs *Level*				*Uphill* vs *Downhill*			
	t^*	n	cluster location	p-values	t^*	n	cluster location	p-values	t^*	n	cluster location	p-values
Unperturbed walking	3.247	4	7.60 – 12.70%	p =0.025	3.227	2	27.29 – 35.22%	p =0.012	3.232	4	3.51– 14.44%	p =0.003
			26.74 –43.56%	p <0.001			53.77 – 62.27%	p =0. 01			22.97 – 44.13%	p <0.001
			59.021 – 63.61%	p =0.029							49.82 – 65.06%	p <0.001
			75.31 – 90.97%	p <0.001							72.44 –95.78%	p <0.001
Slip Recovery	3.601	1	3.31 14.27%	p <0.001	3.619	0	-	-	3.597	2	2.26 11.96%	0.003
											53.27 61.016	0.005
Trip Recovery	3.60	1	3.31-14.27%	p <0.001	3.798	0			3.782	2	0.52 – 10.43%	p <0.001
											67.36 – 72.32%	0.0081

Table S2 Post-hoc Pair-wise comparison (α<0.0167) results of SPM analysis of shown in Fig.3 and Fig.5 in Frontal plane (n= the number of clusters, t*= t-threshold)

Variable	*Downhill* vs *Level*				*Uphill* vs *Level*				*Downhill* vs *Uphill*			
	t^*	n	cluster location	p-values	t^*	n	cluster location	p-values	t^*	n	cluster location	p-values
Unperturbed walking	3.629	0	-	-	3.6124	0	–	–	3.594	1	26.33 –33.76%	0.021
Slip Recovery	3.6801	0	–	–	3.687	0	–	–	3.588	1	77.24 – 87.84%	0.02
Trip Recovery	3.63	1	74.66 – 83.64%	p =0.002	3.796	0	–	–	3.621	2	25.37 – 34.9%	p =0.003
											62. 37 – 88.62%	p <0.001

Table S3. Anteroposterior Perturbation Response (PR_{AP})

Anteroposterior Perturbation Response (PR_{AP})							
						95% Confidence Interval for Difference[b]	
slope	(I) Perturbation	(J) Perturbation	Mean Difference (I-J)	Std. Error	Sig.[b]	Lower Bound	Upper Bound
Level	Slip	Trip	-13.288*	2.121	<.001	-17.764	-8.813
Uphill	Slip	Trip	-6.277*	1.663	.002	-9.785	-2.769
Downhill	Slip	Trip	-2.062*	.798	.019	-3.745	-.379
Based on estimated marginal means							
*. The mean difference is significant at the .05 level.							
b. Adjustment for multiple comparisons: Bonferroni.							

Table S4. Anteroposterior Perturbation Response (PR_{AP})

Anteroposterior Perturbation Response (PR_{AP})							
						95% Confidence Interval for Difference[b]	
Perturbation	(I) slope	(J) slope	Mean Difference (I-J)	Std. Error	Sig.[b]	Lower Bound	Upper Bound
Slip	Level	Uphill	-1.123	1.720	.522	-4.751	2.505
		Downhill	.330	1.186	.784	-2.172	2.833
	Uphill	Level	1.123	1.720	.522	-2.505	4.751
		Downhill	1.453	1.619	.382	-1.963	4.869
	Downhill	Level	-.330	1.186	.784	-2.833	2.172
		Uphill	-1.453	1.619	.382	-4.869	1.963
Trip	Level	Uphill	5.888*	2.306	.021	1.022	10.754
		Downhill	11.556*	2.816	<.001	5.615	17.498
	Uphill	Level	-5.888*	2.306	.021	-10.754	-1.022
		Downhill	5.668*	1.873	.008	1.716	9.621
	Downhill	Level	-11.556	*2.816	<.001	-17.498	-5.615
		Uphill	-5.668*	1.873	.008	-9.621	-1.716

Based on estimated marginal means

*. The mean difference is significant at the .05 level.

b. Adjustment for multiple comparisons: Bonferroni.

Table S5. Mediolateral Perturbation Response (PR_{ML})

Mediolateral Perturbation Response (PR_{ML})							
slope	(I) Perturbation	(J) Perturbation	Mean Difference (I-J)	Std. Error	Sig.[b]	95% Confidence Interval for Difference[b]	
						Lower Bound	Upper Bound
Level	Slip	Trip	-1.015	.743	.189	-2.582	.552
Uphill	Slip	Trip	-1.164*	.348	.004	-1.899	-.430
Downhill	Slip	Trip	-.126	.363	.733	-.892	.640

Based on estimated marginal means

*. The mean difference is significant at the .05 level.

b. Adjustment for multiple comparisons: Bonferroni.

Table S6. Pairwise comparisons of Mediolateral Perturbation Response (PR_{ML})

Mediolateral Perturbation Response (PR_{ML})							
			Mean Difference	Std.		95% Confidence Interval for Difference[a]	
Perturbation	(I) slope	(J) slope	(I-J)	Error	Sig.[a]	Lower Bound	Upper Bound
Slip	Level	Uphill	1.096	.824	.604	-1.093	3.285
		Downhill	1.189	.698	.320	-.664	3.042
	Uphill	Level	-1.096	.824	.604	-3.285	1.093
		Downhill	.093	.588	1.000	-1.470	1.655
	Downhill	Level	-1.189	.698	.320	-3.042	.664
		Uphill	-.093	.588	1.000	-1.655	1.470
Trip	Level	Uphill	.947	.753	.677	-1.053	2.946
		Downhill	2.078	.843	.074	-.161	4.316
	Uphill	Level	-.947	.753	.677	-2.946	1.053
		Downhill	1.131	.516	.128	-.239	2.501
	Downhill	Level	-2.078	.843	.074	-4.316	.161
		Uphill	-1.131	.516	.128	-2.501	.239
Based on estimated marginal means							
a. Adjustment for multiple comparisons: Bonferroni.							

Table S7. Pairwise comparisons of Step Length

Step Length							
			Mean Difference	Std.		95% Confidence Interval for Difference[b]	
Slope	(I) Pert	(J) Pert	(I-J)	Error	Sig.[b]	Lower Bound	Upper Bound
Level	Unperturbed	Slip	.050	.020	.076	-.004	.103
		Trip	.260*	.025	<.001	.192	.327
	Slip	Unperturbed	-.050	.020	.076	-.103	.004
		Trip	.210*	.033	<.001	.122	.298
	Trip	Unperturbed	-.260*	.025	<.001	-.327	-.192
		Slip	-.210*	.033	<.001	-.298	-.122
Uphill	Unperturbed	Slip	.049	.024	.165	-.014	.113
		Trip	.166*	.041	.002	.057	.274
	Slip	Unperturbed	-.049	.024	.165	-.113	.014
		Trip	.116*	.042	.038	.005	.227
	Trip	Unperturbed	-.166*	.041	.002	-.274	-.057
		Slip	-.116*	.042	.038	-.227	-.005
Downhill	Unperturbed	Slip	.073*	.016	<.001	.030	.115
		Trip	.182*	.030	<.001	.103	.260
	Slip	Unperturbed	-.073*	.016	<.001	-.115	-.030
		Trip	.109*	.033	.014	.020	.198
	Trip	Unperturbed	-.182*	.030	<.001	-.260	-.103
		Slip	-.109*	.033	.014	-.198	-.020
Based on estimated marginal means							
*. The mean difference is significant at the .05 level.							
b. Adjustment for multiple comparisons: Bonferroni.							

Table S8. Pairwise comparisons of Step Length

Step Length							
			Mean			95% Confidence Interval for Difference[b]	
Pert	(I) Level	(J) Level	Difference (I-J)	Std. Error	Sig.[b]	Lower Bound	Upper Bound
Unperturbed	Level	Uphill	.001	.014	1.000	-.036	.038
		Downhill	.081*	.013	<.001	.047	.114
	Uphill	Level	-.001	.014	1.000	-.038	.036
		Downhill	.080*	.018	.001	.032	.128
	Downhill	Level	-.081*	.013	<.001	-.114	-.047
		Uphill	-.080*	.018	.001	-.128	-.032
Slip	Level	Uphill	.001	.026	1.000	-.068	.070
		Downhill	.104*	.030	.008	.026	.182
	Uphill	Level	-.001	.026	1.000	-.070	.068
		Downhill	.103*	.030	.010	.023	.184
	Downhill	Level	-.104*	.030	.008	-.182	-.026
		Uphill	-.103*	.030	.010	-.184	-.023
Trip	Level	Uphill	-.093	.044	.155	-.211	.025
		Downhill	.003	.038	1.000	-.097	.103
	Uphill	Level	.093	.044	.155	-.025	.211
		Downhill	.096	.047	.170	-.029	.221
	Downhill	Level	-.003	.038	1.000	-.103	.097
		Uphill	-.096	.047	.170	-.221	.029
Based on estimated marginal means							
*. The mean difference is significant at the .05 level.							
b. Adjustment for multiple comparisons: Bonferroni.							

Table S9. Pairwise comparisons of Step Width

Step Width							
	(I)	(J)	Mean			95% Confidence Interval for Difference[b]	
Slope	Perturbation	Perturbation	Difference (I-J)	Std. Error	Sig.[b]	Lower Bound	Upper Bound
Level	Unperturbed	Slip	-.072*	.013	<.001	-.105	-.038
		Trip	-.068*	.008	<.001	-.089	-.046
	Slip	Unperturbed	.072*	.013	<.001	.038	.105
		Trip	.004	.012	1.000	-.027	.035
	Trip	Unperturbed	.068*	.008	<.001	.046	.089
		Slip	-.004	.012	1.000	-.035	.027
Uphill	Unperturbed	Slip	-.059*	.014	.002	-.095	-.022
		Trip	-.057*	.013	.001	-.091	-.022
	Slip	Unperturbed	.059*	.014	.002	.022	.095
		Trip	.002	.013	1.000	-.031	.036
	Trip	Unperturbed	.057*	.013	.001	.022	.091
		Slip	-.002	.013	1.000	-.036	.031
Downhill	Unperturbed	Slip	-.024	.010	.096	-.052	.003
		Trip	-.037*	.011	.008	-.065	-.009
	Slip	Unperturbed	.024	.010	.096	-.003	.052
		Trip	-.012	.008	.422	-.034	.009
	Trip	Unperturbed	.037*	.011	.008	.009	.065
		Slip	.012	.008	.422	-.009	.034
Based on estimated marginal means							
*. The mean difference is significant at the .05 level.							
b. Adjustment for multiple comparisons: Bonferroni.							

Table S10. Pairwise comparisons of Step Width

Step Width							
Perturbation	(I) Slope	(J) Slope	Mean Difference (I-J)	Std. Error	Sig.[a]	95% Confidence Interval for Difference[a] Lower Bound	Upper Bound
Unperturbed	Level	Uphill	-.018*	.007	.042	-.036	-.001
		Downhill	-.028	.012	.095	-.060	.004
	Uphill	Level	.018*	.007	.042	.001	.036
		Downhill	-.010	.012	1.000	-.042	.022
	Downhill	Level	.028	.012	.095	-.004	.060
		Uphill	.010	.012	1.000	-.022	.042
Slip	Level	Uphill	-.005	.013	1.000	-.040	.029
		Downhill	.019	.016	.711	-.022	.060
	Uphill	Level	.005	.013	1.000	-.029	.040
		Downhill	.024	.015	.392	-.016	.065
	Downhill	Level	-.019	.016	.711	-.060	.022
		Uphill	-.024	.015	.392	-.065	.016
Trip	Level	Uphill	-.007	.010	1.000	-.033	.018
		Downhill	.002	.013	1.000	-.032	.037
	Uphill	Level	.007	.010	1.000	-.018	.033
		Downhill	.010	.015	1.000	-.030	.050
	Downhill	Level	-.002	.013	1.000	-.037	.032
		Uphill	-.010	.015	1.000	-.050	.030
Based on estimated marginal means							
a. Adjustment for multiple comparisons: Bonferroni.							

Table S11. Pairwise comparisons of integrated WBAM (iWBAM) in sagittal plane

Sagittal iWBAM							
			Mean Difference			95% Confidence Interval for Difference[b]	
Slope	(I) Pert	(J) Pert	(I-J)	Std. Error	Sig.[b]	Lower Bound	Upper Bound
Level	Unperturbed	Slip	-.063*	.011	<.001	-.092	-.034
		Trip	-.060*	.008	<.001	-.081	-.040
	Slip	Unperturbed	.063*	.011	<.001	.034	.092
		Trip	.003	.010	1.000	-.025	.030
	Trip	Unperturbed	.060*	.008	<.001	.040	.081
		Slip	-.003	.010	1.000	-.030	.025
Uphill	Unperturbed	Slip	-.052*	.012	.001	-.084	-.020
		Trip	-.050*	.012	.002	-.081	-.019
	Slip	Unperturbed	.052*	.012	.001	.020	.084
		Trip	.002	.011	1.000	-.027	.032
	Trip	Unperturbed	.050*	.012	.002	.019	.081
		Slip	-.002	.011	1.000	-.032	.027
Downhill	Unperturbed	Slip	-.021	.009	.098	-.045	.003
		Trip	-.032*	.009	.009	-.056	-.008
	Slip	Unperturbed	.021	.009	.098	-.003	.045
		Trip	-.011	.007	.391	-.029	.007
	Trip	Unperturbed	.032*	.009	.009	.008	.056
		Slip	.011	.007	.391	-.007	.029
Based on estimated marginal means							
*. The mean difference is significant at the .05 level.							
b. Adjustment for multiple comparisons: Bonferroni.							

Table S12. Pairwise comparisons of integrated WBAM (iWBAM) in sagittal plane

Sagittal iWBAM							
			Mean Difference			95% Confidence Interval for Difference[b]	
Pert	(I) Slope	(J) Slope	(I-J)	Std. Error	Sig.[b]	Lower Bound	Upper Bound
Unperturbed	Level	Uphill	-.468*	.085	<.001	-.694	-.243
		Downhill	.208*	.063	.012	.042	.374
	Uphill	Level	.468*	.085	<.001	.243	.694
		Downhill	.676*	.060	<.001	.518	.834
	Downhill	Level	-.208*	.063	.012	-.374	-.042
		Uphill	-.676*	.060	<.001	-.834	-.518
Slip	Level	Uphill	-.121	.170	1.000	-.573	.331
		Downhill	.290*	.085	.010	.066	.514
	Uphill	Level	.121	.170	1.000	-.331	.573
		Downhill	.411	.158	.056	-.009	.831
	Downhill	Level	-.290*	.085	.010	-.514	-.066
		Uphill	-.411	.158	.056	-.831	.009
Trip	Level	Uphill	-.233	.136	.310	-.593	.127
		Downhill	.893*	.150	<.001	.495	1.292
	Uphill	Level	.233	.136	.310	-.127	.593
		Downhill	1.126*	.119	<.001	.811	1.442
	Downhill	Level	-.893*	.150	<.001	-1.292	-.495
		Uphill	-1.126*	.119	<.001	-1.442	-.811
Based on estimated marginal means							
*. The mean difference is significant at the .05 level.							
b. Adjustment for multiple comparisons: Bonferroni.							

Table S13. Pairwise comparisons of Range of WBAM in sagittal plane

Sagittal Range							
			Mean Difference			95% Confidence Interval for Difference[b]	
Slope	(I) Pert	(J) Pert	(I-J)	Std. Error	Sig.[b]	Lower Bound	Upper Bound
Level	Unperturbed	Slip	-.011*	.004	.023	-.021	-.001
		Trip	-.023*	.004	<.001	-.034	-.012
	Slip	Unperturbed	.011*	.004	.023	.001	.021
		Trip	-.012	.006	.181	-.027	.004
	Trip	Unperturbed	.023*	.004	<.001	.012	.034
		Slip	.012	.006	.181	-.004	.027
Uphill	Unperturbed	Slip	-.002	.004	1.000	-.014	.009
		Trip	-.021*	.004	<.001	-.031	-.010
	Slip	Unperturbed	.002	.004	1.000	-.009	.014
		Trip	-.018*	.004	<.001	-.028	-.009
	Trip	Unperturbed	.021*	.004	<.001	.010	.031
		Slip	.018*	.004	<.001	.009	.028
Downhill	Unperturbed	Slip	-.008	.004	.250	-.019	.003
		Trip	-.011*	.003	.011	-.020	-.002
	Slip	Unperturbed	.008	.004	.250	-.003	.019
		Trip	-.003	.005	1.000	-.015	.009
	Trip	Unperturbed	.011*	.003	.011	.002	.020
		Slip	.003	.005	1.000	-.009	.015
Based on estimated marginal means							
*. The mean difference is significant at the .05 level.							
b. Adjustment for multiple comparisons: Bonferroni.							

Table S14. Pairwise comparisons of Range of WBAM in sagittal plane

Sagittal Range							
			Mean Difference	Std.		95% Confidence Interval for Difference[b]	
Pert	(I) Slope	(J) Slope	(I-J)	Error	Sig.[b]	Lower Bound	Upper Bound
Unperturbed	level	Uphill	-.015*	.003	<.001	-.024	-.006
		Downhill	.004	.003	.291	-.002	.011
	Uphill	Level	.015*	.003	<.001	.006	.024
		Downhill	.019*	.003	<.001	.011	.028
	Downhill	Level	-.004	.003	.291	-.011	.002
		Uphill	-.019*	.003	<.001	-.028	-.011
Slip	level	Uphill	-.006	.006	.900	-.021	.009
		Downhill	.008	.003	.060	.000	.016
	Uphill	Level	.006	.006	.900	-.009	.021
		Downhill	.014	.006	.091	-.002	.030
	Downhill	Level	-.008	.003	.060	-.016	.000
		Uphill	-.014	.006	.091	-.030	.002
Trip	level	Uphill	-.013*	.004	.016	-.023	-.002
		Downhill	.016*	.004	.005	.005	.028
	Uphill	Level	.013*	.004	.016	.002	.023
		Downhill	.029*	.005	<.001	.017	.041
	Downhill	Level	-.016*	.004	.005	-.028	-.005
		Uphill	-.029*	.005	<.001	-.041	-.017
Based on estimated marginal means							
*. The mean difference is significant at the .05 level.							
b. Adjustment for multiple comparisons: Bonferroni.							

Table S15. Pairwise comparisons of integrated WBAM (iWBAM) in frontal plane

Frontal iWBAM							
			Mean Difference	Std.		95% Confidence Interval for Difference[b]	
Slope	(I) Pert	(J) Pert	(I-J)	Error	Sig.[b]	Lower Bound	Upper Bound
Level	Unperturbed	Slip	-.324*	.061	<.001	-.487	-.161
		Trip	-.185	.090	.166	-.423	.054
	Slip	Unperturbed	.324*	.061	<.001	.161	.487
		Trip	.140	.099	.531	-.124	.403
	Trip	Unperturbed	.185	.090	.166	-.054	.423
		Slip	-.140	.099	.531	-.403	.124
Uphill	Unperturbed	Slip	-.256*	.091	.036	-.498	-.014
		Trip	-.279*	.072	.004	-.469	-.089
	Slip	Unperturbed	.256*	.091	.036	.014	.498
		Trip	-.023	.084	1.000	-.246	.200
	Trip	Unperturbed	.279*	.072	.004	.089	.469
		Slip	.023	.084	1.000	-.200	.246
Downhill	Unperturbed	Slip	-.144	.067	.139	-.323	.034
		Trip	-.070	.079	1.000	-.278	.139
	Slip	Unperturbed	.144	.067	.139	-.034	.323
		Trip	.075	.043	.289	-.038	.188
	Trip	Unperturbed	.070	.079	1.000	-.139	.278
		Slip	-.075	.043	.289	-.188	.038
Based on estimated marginal means							
*. The mean difference is significant at the .05 level.							
b. Adjustment for multiple comparisons: Bonferroni.							

Table S16. Pairwise comparisons of integrated WBAM (iWBAM) in frontal plane

Frontal iWBAM							
			Mean Difference	Std.		95% Confidence Interval for Difference[b]	
Pert	(I) Slope	(J) Slope	(I-J)	Error	Sig.[b]	Lower Bound	Upper Bound
Unperturbed	Level	Uphill	-.226*	.032	<.001	-.311	-.140
		Downhill	-.035	.065	1.000	-.209	.139
	Uphill	Level	.226*	.032	<.001	.140	.311
		Downhill	.191	.074	.058	-.005	.387
	Downhill	Level	.035	.065	1.000	-.139	.209
		Uphill	-.191	.074	.058	-.387	.005
Slip	Level	Uphill	-.157	.093	.330	-.405	.090
		Downhill	.145	.061	.090	-.017	.307
	Uphill	Level	.157	.093	.330	-.090	.405
		Downhill	.302*	.108	.038	.015	.590
	Downhill	Level	-.145	.061	.090	-.307	.017
		Uphill	-.302*	.108	.038	-.590	-.015
Trip	Level	Uphill	-.320*	.100	.015	-.585	-.055
		Downhill	.080	.106	1.000	-.201	.361
	Uphill	Level	.320*	.100	.015	.055	.585
		Downhill	.400*	.082	<.001	.181	.619
	Downhill	Level	-.080	.106	1.000	-.361	.201
		Uphill	-.400*	.082	<.001	-.619	-.181
Based on estimated marginal means							
*. The mean difference is significant at the .05 level.							
b. Adjustment for multiple comparisons: Bonferroni.							

Table S17. Pairwise comparisons of Range of WBAM in frontal plane

Frontal Range							
			Mean Difference	Std.		95% Confidence Interval for Difference[b]	
Slope	(I) Pert	(J) Pert	(I-J)	Error	Sig.[b]	Lower Bound	Upper Bound
Level	Unperturbed	Slip	-.009*	.002	<.001	-.014	-.004
		Trip	-.004	.003	.835	-.013	.005
	Slip	Unperturbed	.009*	.002	<.001	.004	.014
		Trip	.005	.004	.698	-.006	.016
	Trip	Unperturbed	.004	.003	.835	-.005	.013
		Slip	-.005	.004	.698	-.016	.006
Uphill	Unperturbed	Slip	-.007	.003	.133	-.015	.002
		Trip	-.005	.003	.215	-.012	.002
	Slip	Unperturbed	.007	.003	.133	-.002	.015
		Trip	.002	.003	1.000	-.005	.009
	Trip	Unperturbed	.005	.003	.215	-.002	.012
		Slip	-.002	.003	1.000	-.009	.005
Downhill	Unperturbed	Slip	-.005	.003	.269	-.012	.002
		Trip	-.002	.003	1.000	-.010	.005
	Slip	Unperturbed	.005	.003	.269	-.002	.012
		Trip	.003	.002	.694	-.003	.008
	Trip	Unperturbed	.002	.003	1.000	-.005	.010
		Slip	-.003	.002	.694	-.008	.003
Based on estimated marginal means							
*. The mean difference is significant at the .05 level.							
b. Adjustment for multiple comparisons: Bonferroni.							

Table S18. Pairwise comparisons of Range of WBAM in frontal plane

Frontal Range							
			Mean Difference	Std.		95% Confidence Interval for Difference[b]	
Pert	(I) Slope	(J) Slope	(I-J)	Error	Sig.[b]	Lower Bound	Upper Bound
Unperturbed	Level	Uphill	-.011*	.002	<.001	-.016	-.006
		Downhill	.001	.002	1.000	-.005	.006
	Uphill	Level	.011*	.002	<.001	.006	.016
		Downhill	.011	.003	.007	.003	.020
	Downhill	Level	-.001	.002	1.000	-.006	.005
		Uphill	-.011*	.003	.007	-.020	-.003
Slip	Level	Uphill	-.009*	.003	.039	-.017	.000
		Downhill	.005	.002	.075	.000	.010
	Uphill	Level	.009*	.003	.039	.000	.017
		Downhill	.013	.004	.005	.004	.023
	Downhill	Level	-.005	.002	.075	-.010	.000
		Uphill	-.013*	.004	.005	-.023	-.004
Trip	Level	Uphill	-.012*	.003	.005	-.020	-.003
		Downhill	.002	.003	1.000	-.006	.011
	Uphill	Level	.012*	.003	.005	.003	.020
		Downhill	.014*	.003	<.001	.007	.021
	Downhill	Level	-.002	.003	1.000	-.011	.006
		Uphill	-.014*	.003	<.001	-.021	-.007
Based on estimated marginal means							
*. The mean difference is significant at the .05 level.							
b. Adjustment for multiple comparisons: Bonferroni.							
