# Supplementary material for: Recovering whole-body angular momentum and margin of stability after treadmill-induced perturbations during sloped walking in healthy young adults
Source: Sci Rep. 2024 Feb 23;14:4421. doi: 10.1038/s41598-024-54890-4 (PMC10884438; doi:10.1038/s41598-024-54890-4)
Supplement: Supplementary file 1 — Supplementary Information 1. [file 41598_2024_54890_MOESM1_ESM.zip › Data_Paper/Readme.pdf]

About the data set

This data set was created as a part of a comprehensive study to investigate reactive recovery responses following treadmill-induced perturbations on both level and sloped surfaces. The investigation featured three distinct slope angles: downhill: -8°; level: 0°; uphill: +8°. The data was collected from 18 participants including 9 females and 9 males.

Included Data:

The dataset encompasses *TRCs* motion files, a scaled OSIM model, *BodyKinematics*, and *gaitEvents* files providing information on heel strike and toe-off locations for each trial for each participant. Motion data collection frequency is 100 Hz and data is presented in millimeters. All data is conveniently formatted in MATLAB data format (MAT files).

Trials:

We administered 2 different perturbations (slip and trip-like) on 3 different slopes (level, downhill, and uphill). Therefore, a total of 162 trials were collected in total. , with each subject participating in nine different trials:

1. **W2_Down_Slip:** Slip perturbation on downhill surface
2. **W2_Down_Trip:** Trip perturbation on downhill surface
3. **W2_Down_i:** Unperturbed walking on downhill surface
4. **W2_Normal:** Unperturbed walking on level surface
5. **W2_Slip:** Slip perturbation on level surface
6. **W2_Trip:** Trip perturbation on level surface
7. **W2_Up_Slip:** Slip perturbation on uphill surface
8. **W2_Up_Trip:** Trip perturbation on uphill surface
9. **W2_Up_i:** Unperturbed walking on uphill surface

Note: The presence of "W2" and "i" in some of the trial names and the order of trials in the files are because of the fact that this dataset is a part of a broader dataset in which we had various walking speeds and other conditions.

Marker set:

The marker set used in the data set is shown in the figure below. This marker set is compatible with HBM-gait model in the CAREN system. Includes:

- Torso: T10,C7,JN,XIPH
- Pelvis: LASIS, RASIS, LPSIS, RPSIS
- Legs: RLTHI, LLTHI, RLSHA, LLSHA, RLEK, LLEK, RMEK, LMEK
- Left and right feet: RMM, LMM, RLM, LLM, RHEE, LHEE, RMT2, LMT2, RMT5, LMT5

❖ Note: the unit in the TRCs file is in mm and should be converted to m for calculations.

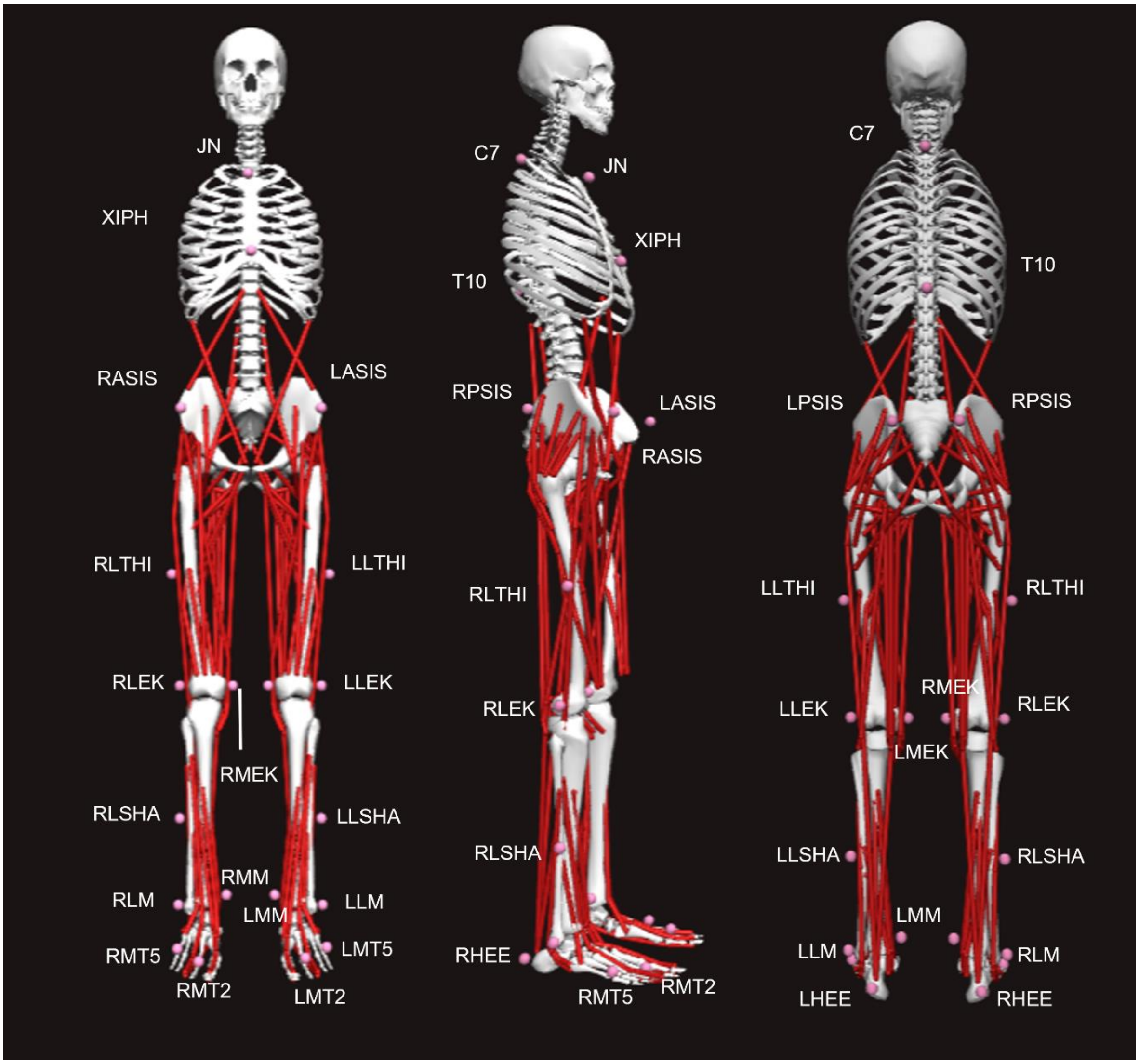

Front, side, and rear view of the marker set used in the data set

Trials Info File: Trials_info.xlsx

This file provides information about subjects and the needed information for batch-processing of the data. A spreadsheet, comprising 11 sheets, is provided. The "Info" sheet contains subjects' details, such as sex, height, mass, and BMI. Additionally, the "Trials_WBAM" sheet offers inputs for the *RunWBAM.m* code, while the remaining nine sheets include inputs for *Run_MoS_unperturbed.m* and *Run_MoS_Perturbed.m* codes, facilitating MoS calculations for unperturbed and perturbed trials. These inputs include:

Column 1 (Index): Represents the trial index (i=9).

Column 2 (Perturbation Step Index): Indicates the step index in which perturbation was applied.

Column 3 (Recovery Step Index): Represents the index of the first recovery step (Rec1) taken by the left leg following the perturbation.

For example, if the perturbation index for a trial is 4 it means that perturbation was applied at the 4th right heel strike in that recorded trial. Similarly, if the recovery index is 4, it means that the 4th left leg's step is the first recovery step (Rec1).

Column 4 (n): Specifies the number of steps following the perturbation that are of interest. For perturbation trials, this value is set to 3, indicating the calculation of MoS at heel strike for 3 steps taken by the right leg following the perturbation and 3 steps taken by the left leg following the perturbation step making 6 recovery steps in total for the calculation of PR (Rec1, Rec2, Rec3, Rec4, Rec5, Rec6).

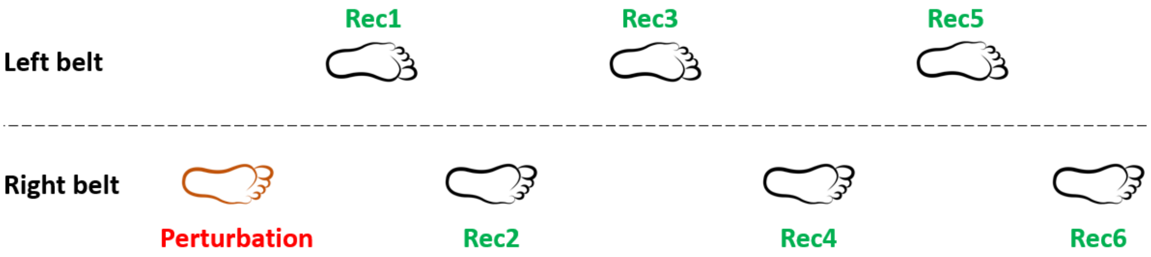

Note: For unperturbed trials, perturbation index, recovery index, and n were set as 5 and 9, meaning that *MoS_Unperturbed.m* code calculates the MoS starting from the 5th step to ensure steady state condition, and calculates the 9 right and 9 left steps after that, adding

up to 20 unperturbed steps in total. Then calculates and saves the mean and standard deviation of these 20 steps to be used in the perturbation response calculation.

Coordinate system:

X: Anteroposterior, +X is forward

Y: Vertical, +Y is upward

Z: Mediolateral, +Z is to the right

Contact Information:

For any inquiries related to this dataset, please reach out to Shabnam Shokouhi via email at sshokouhi@student.unimelb.edu.au.
