# Supplementary material for: Recovering whole-body angular momentum and margin of stability after treadmill-induced perturbations during sloped walking in healthy young adults
Source: Sci Rep. 2024 Feb 23;14:4421. doi: 10.1038/s41598-024-54890-4 (PMC10884438; doi:10.1038/s41598-024-54890-4)
Supplement: Supplementary file 1 — Supplementary Information 1. [file 41598_2024_54890_MOESM1_ESM.zip › Data_Paper/Results/Figures/Figure2.pdf]

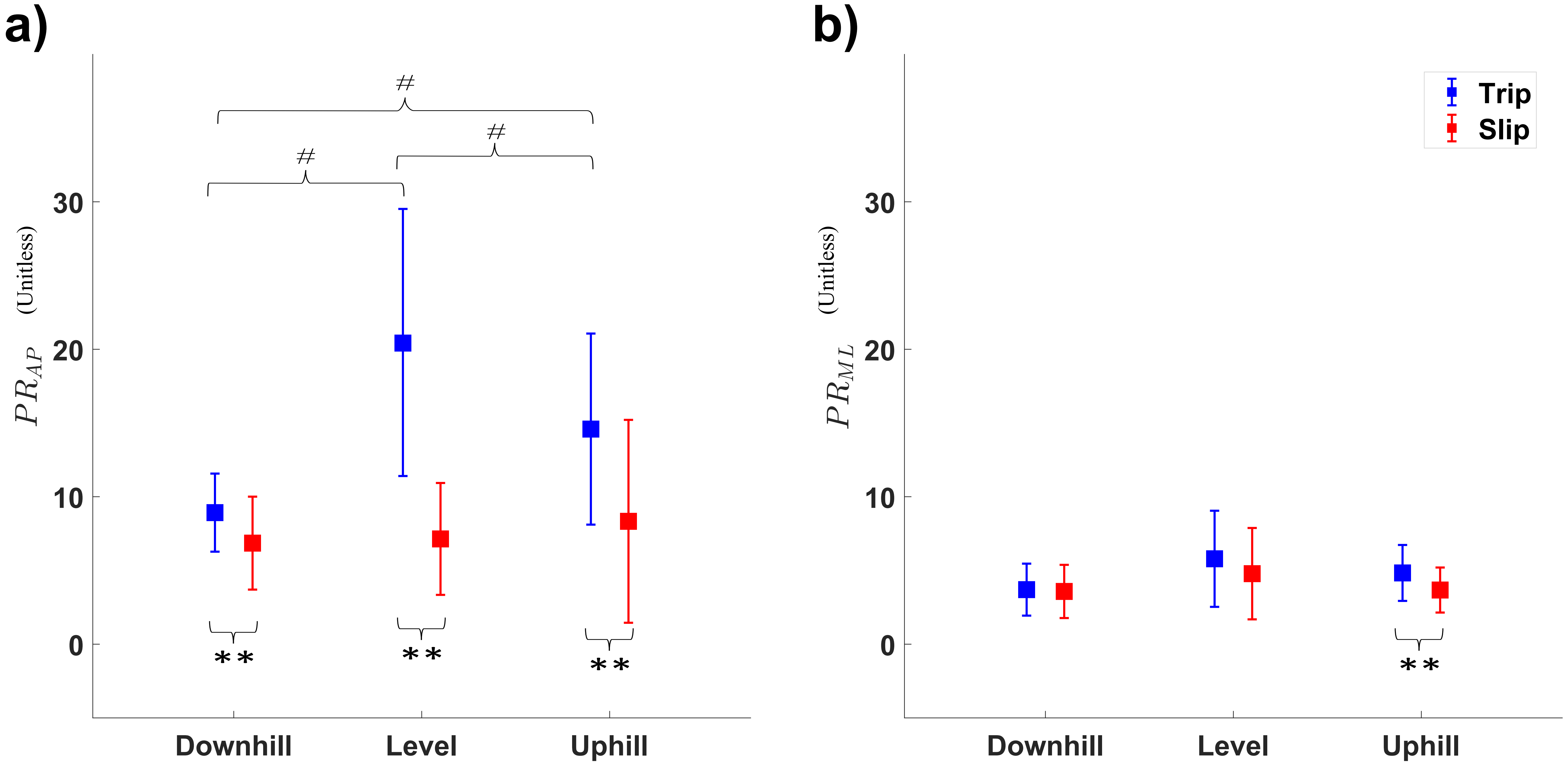

a)
b)
PR_{AP} (Unitless)
PR_{ML} (Unitless)
0
10
20
30
#
#
#
**
**
**
**
Downhill
Level
Uphill
Trip
Slip
