# Supplementary material for: Recovering whole-body angular momentum and margin of stability after treadmill-induced perturbations during sloped walking in healthy young adults
Source: Sci Rep. 2024 Feb 23;14:4421. doi: 10.1038/s41598-024-54890-4 (PMC10884438; doi:10.1038/s41598-024-54890-4)
Supplement: Supplementary file 1 — Supplementary Information 1. [file 41598_2024_54890_MOESM1_ESM.zip › Data_Paper/Results/Figures/Figure3.pdf]

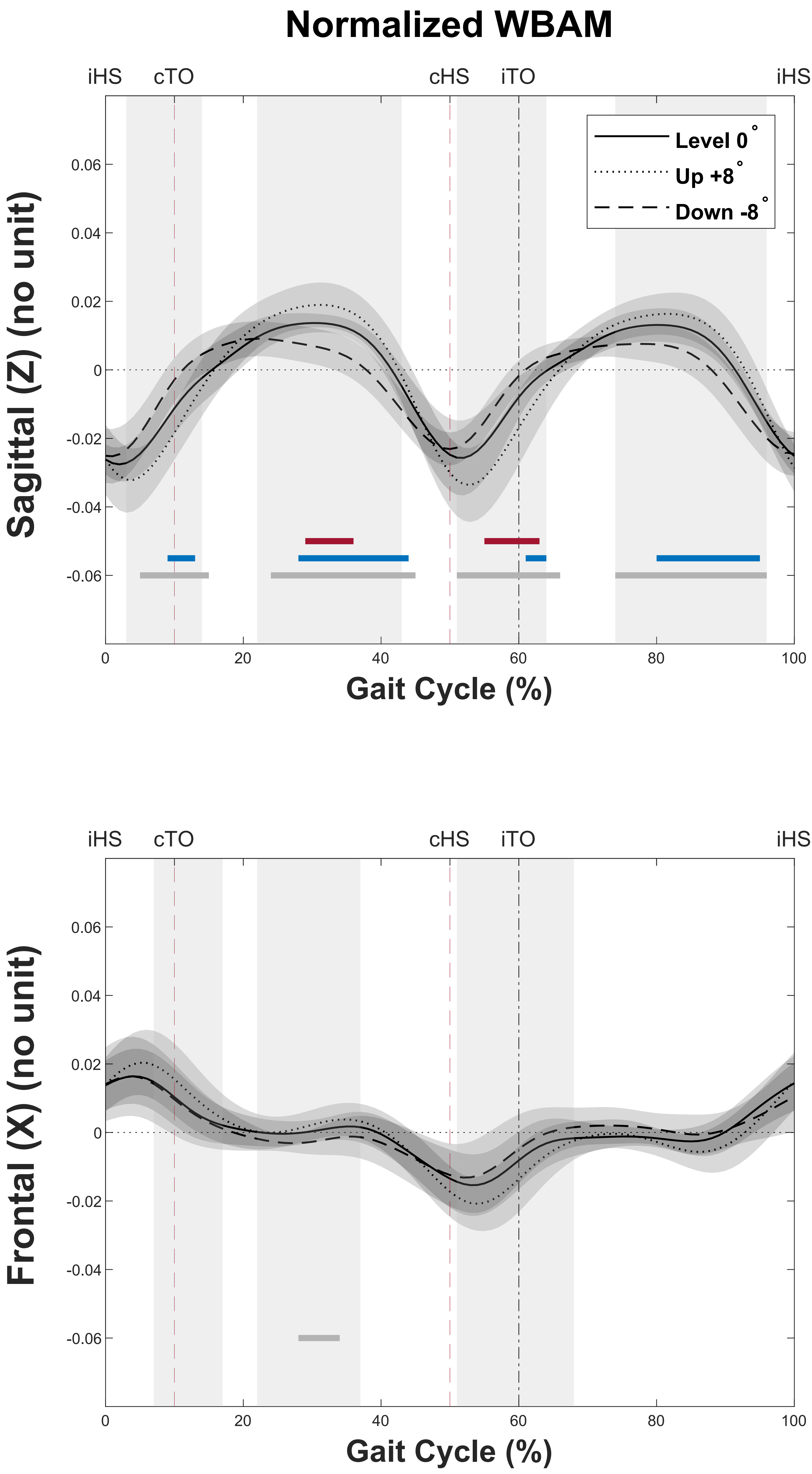

Normalized WBAM
iHS
cTO
cHS
iTO
iHS
Level 0°
Up +8°
Down -8°
Sagittal (Z) (no unit)
0.06
0.04
0.02
0
-0.02
-0.04
-0.06
0
20
40
60
80
100
Gait Cycle (%)
iHS
cTO
cHS
iTO
iHS
Frontal (X) (no unit)
0.06
0.04
0.02
0
-0.02
-0.04
-0.06
0
20
40
60
80
100
Gait Cycle (%)
